# Supplementary material for: VUStruct: A compute pipeline for high throughput and personalized structural biology
Source: PLoS Comput Biol. 2026 May 4;22(5):e1014183. doi: 10.1371/journal.pcbi.1014183 (PMC13160433; doi:10.1371/journal.pcbi.1014183)
Supplement: S2 Text — (PDF) [file pcbi.1014183.s002.pdf]

S2 VUStruct Supplemental Information

Structure Selection

As noted in the main paper, VUStruct mines experimental structures from the PDB and aligns them to current transcripts via the SIFTS database. SwissModel and ModBase homology models are integrated. For mutations that impact canonical transcripts, AlphaFold models are added to the set of representative structures. The final structure selections minimize redundancy and maximize diversity of experimental techniques, variant-coverage, model confidence and experimental quality metrics. Multimeric complexes are also prioritized in this process.

To implement this vision under the hood, the "psb_plan" python module interrogates the mentioned model repositories to build the widest universe of structures for consideration. Following quality checks, an iterative and pair-wise structure comparison is performed through which weaker structures are discarded. For each case, this is all logged by the "plan" module, along with a structure selection report with counts, which is created before pipeline launch

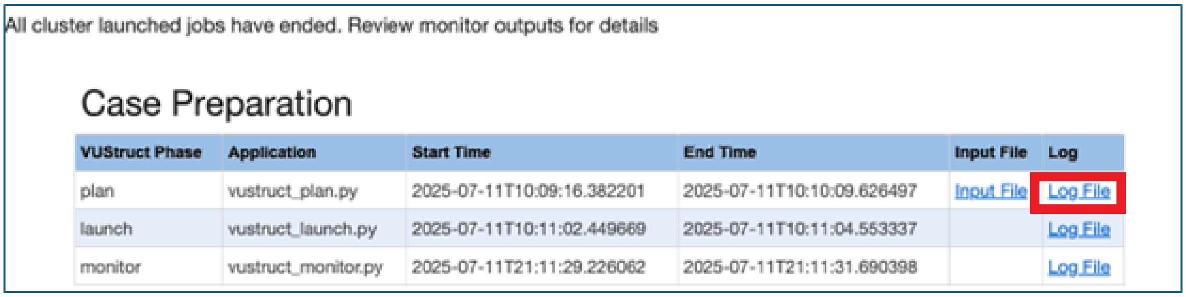

All cluster launched jobs have ended. Review monitor outputs for details

Case Preparation

VUStruct Phase	Application	Start Time	End Time	Input File	Log
plan	vustruct_plan.py	2025-07-11T10:09:16.382201	2025-07-11T10:10:09.626497	Input File	Log File
launch	vustruct_launch.py	2025-07-11T10:11:02.449669	2025-07-11T10:11:04.553337		Log File
monitor	vustruct_monitor.py	2025-07-11T21:11:29.226062	2025-07-11T21:11:31.690398		Log File

Fig S2. The log is available for review or download in the "log" column at the top right of the case landing page

The psb_plan.py module, located at the Github site, loads each structure's .cif or .pdb file and calls set_alignment_profile() to label it according to resolved residues. The set_alignment_profile() method will label it as either "Yes," meaning it's fully analyzable (residue is resolved fully); "Maybe," meaning it's suitable for limited visualization (some near-in-sequence residues are resolved); or "No," meaning it is neither resolved or near other resolved residues in sequence. This drops the structure from further consideration.

For model structures, set_alignment_profile() is called after a check of transcript to chain alignment. Small deviations at individual sequence positions are tolerated. To locate PDB structures for a given uniprot transcript, pre-loaded SQL tables containing results from the SIFTS REST API are queried. The SIFTS-guided transcript-to-chain alignment that follows. For alignment of canonical transcripts to PDB residues, the detailed per-residue XML SIFTS

files are used. For non-canonical transcript alignments, the SIFTS-RESTAPI alignment ranges are used with care to ensure that SFITS-expected template identities are achieved. (These are regularly updated for all human transcript isoforms in the VUStruct SQL database.). The product of alignment is an in-memory dictionary (mapping) of transcript offsets to PDB Residue IDs in Biopython format.

The multimeric state of structures and models is approximated by the chain count in the deposition or model. Solution Scattering structures lack side chains and these are eliminated from consideration.

During pairwise structural comparisons, a non-random heuristic is employed to whittle down the full structure list to reduce redundancy. Reviewing the drop_df_row() calls in the code reveals that a structure is dropped from a pair being compared, when they **both share**:

- experimental or modeling method, and
- multimeric state, and
- PDB template (for models)

but differ as one of the two structures:

- resolves the residue (analyzable=YES) and the other does not (No or Maybe), or
- models a longer chain than the other
- one is swiss and the other is modbase.

When two structures seem "identical" by these criteria, we simply drop a structure based on the lower chain ID or structural ID to differentiate them. When run at the back end (vs web input form), structures that are user supplied or listed in the configuration files are never dropped.

Finally, a pass through the remaining structures is performed, in which low-template-identity models are dropped when other structure types are available. See calls to drop_df_low_identity_rows() in the psb_plan.py source.

Emphasis: All structure decisions are transparently reported in the plan log. Below is an excerpt from the plan logfor the CDG10 variant (from the sample case used in the main text screenshots):

```
11 CDH10   10:09:31 INFO [     psb_plan.py:1582] Dropping ci_df duplicate row 2 because
Q9Y6N8_55_594_7a7d.1.M.M is shorter swissmodel vs Q9Y6N8_55_598_7a7d.1.B.B

 11 CDH10   10:09:31 INFO [     psb_plan.py:1582] Dropping ci_df duplicate row 5 because
Q9Y6N8_55_598_7a7d.1.C.C is same length, but greater than or same chain ID vs
Q9Y6N8_55_598_7a7d.1.B.B

 11 CDH10   10:09:31 INFO [     psb_plan.py:1582] Dropping ci_df duplicate row 5 because
Q9Y6N8_55_594_7a7d.1.H.H is shorter swissmodel vs Q9Y6N8_55_598_7a7d.1.B.B

 11 CDH10   10:09:31 INFO [     psb_plan.py:1582] Dropping ci_df duplicate row 5 because
Q9Y6N8_55_589_7a7d.1.A.A is shorter swissmodel vs Q9Y6N8_55_598_7a7d.1.B.B

 11 CDH10   10:09:31 INFO [     psb_plan.py:1791] Dropping of row 5 because
Q9Y6N8_55_598_7a7d.1.B Low template_identity Models are not needed

 11 CDH10   10:09:31 INFO [     psb_plan.py:1791] Dropping of row 11 because ENSP00000264463.4_3
Low template_identity Models are not needed

 11 CDH10   10:09:31 INFO [     psb_plan.py:1791] Dropping of row 12 because ENSP00000264463.4_2
Low template_identity Models are not needed

 11 CDH10   10:09:31 INFO [     psb_plan.py:1791] Dropping of row 14 because ENSP00000264463.4_5
Low template_identity Models are not needed
```

At the end of the log VUStruct outputs a summary for each variant, showing counts of structures retained and dropped, as well as a compute job count.

Structure Report

	gene	refseq	mutation	unp	retained	dropped	jobs
0	TCF3	NM_003200.3	H85Y	P15923-1	2	9	10
1	TCF3	NM_001136139.2	H85Y	P15923-2	0	4	0
2	AP3D1	NM_003938.6	V699M	O14617-1	4	7	17
3	AP3D1	NM_001261826.1	V699M	O14617-5	1	7	3
4	AP3D1	NM_003938.6	A299T	O14617-1	3	7	15
5	AP3D1	NM_001261826.1	A299T	O14617-5	1	7	5
6	GUCA1C	NM_005459.3	I168M	O95843-1	5	3	24
7	GUCA1C	NM_005459.3	G33D	O95843-1	6	2	27
8	BMP2K	NM_198892.1	P716S	Q9NSY1-1	1	11	7
9	CDH10	NM_006727.4	T159M	Q9Y6N8	9	9	36
10	CR2	NM_001877.4	F708L	P20023-1	2	21	10
11	CR2	NM_001006658.2	F767L	P20023-3	1	6	3
12	DES	NM_001927.3	A213V	P17661	4	7	22
13	ESR1	NM_000125.3	T76S	P03372-1	1	778	7
14	HFE	NM_000410.3	V59M	Q30201-1	6	5	24
15	HFE	NM_139003.2	V59M	Q30201-10	4	4	17
16	HFE	NM_139006.2	V59M	Q30201-3	3	2	12
17	HFE	NM_139009.2	V36M	Q30201-5	3	3	12
18	HFE	NM_139004.2	V59M	Q30201-7	3	3	12
19	LIMK1	NM_002314.3	H614Y	P53667-1	9	26	34
20	LIMK1	NM_001204426.1	H580Y	P53667-4	4	10	18
21	MBD5	NM_018328.4	P298L	Q9P267-1	2	5	10
22	MBD5	NA	P298L	Q9P267-2	0	3	0
23	SERPINA6	NM_001756.3	D269G	P08185	8	10	36
24	WDR41	NM_018268.3	H322R	Q9HAD4-1	9	7	34
25	WDR41	NA	H267R	Q9HAD4-2	7	7	26

We do not claim this system to be optimal in any quantified or quantifiable sense. It has simply evolved organically over the past decade, in response to user requests concomitant with a revolutionary proliferation in the number and variety of deposited structures and models.

The algorithm is not static, and this description is superceded by the actual plan.py code at the GitHub repository. In the algorithm's current form, we sometimes encounter variants for which too many structures are selected (i.e web browsers can no longer display all the graphics, and/or no obvious insight is gained from the proliferation). This challenges arises from growing homology model counts as deposited candidate templates increase. CryoEM Multimers also take graphics card memory, which we should factor in as well.

However, the current selection locates essentially the same models we would find with manual search.

Custom Models and Selection Customization

While today's web input form does not allow structure selection customization, the back-end pipeline supports customization of the structure selection process in two ways.

In VUStruct, back-end configuration options parameterize and regulate a range of pipeline behaviors and provide paths to data resources. The configuration is customizable in a hierarchy. While the global.config file is satisfactory for most applications, there is a userid.config file that can override global options on a per-user basis. At the next level, one can create a caseid.config file, overriding both global and user settings for a specific variant set. To ensure that one or more specific PDB IDs are <u>never</u> discarded during structure selection, the KeepPDB= flag can be set to a comma-delimited list of the IDs to always retain.

Alternatively, for any variant formatted in a vustruct.csv input file, an optional 'user_model' column can be added to each variant input row to specify a filename. While a per-variant upload feature in the UI might be unwieldy, we could imagine expanding the input forms to allow this. Alternately, we could imagine allowing general URIs in this column and thus enable the pipeline to retrieve .cif files from user-provided resources.

As we contemplate improvements to support back-end flexibility in the web input forms and file formats, we are committed to supporting any investigator's inquiry to run the pipeline with custom model(s). To support some investigations, we have even quickly created bespoke versions of the structure selection python "plan" module.

We hope to soon integrate Boltz-2 structure predictions. This should mitigate the growing problem that Alphafold 2 models are neither being refreshed (such that transcript re-assignments result in lack of Alphafold 2 structure coverage) nor retrained on updated experimental depositions.
