# Supplementary material for: VUStruct: A compute pipeline for high throughput and personalized structural biology
Source: PLoS Comput Biol. 2026 May 4;22(5):e1014183. doi: 10.1371/journal.pcbi.1014183 (PMC13160433; doi:10.1371/journal.pcbi.1014183)
Supplement: S3 Text — (PDF) [file pcbi.1014183.s003.pdf]

S3 VUStruct Supplemental Information

Runtime Expectations

To serve the standing weekly Undiagnosed Clinic Disease Network clinical meetings at Vanderbilt each **Friday**, the pipeline receives curated input variants in genomic coordinates on the prior **Tuesday** and completes most calculations in time for the structural biology consultant(s) to populate the case summary worksheet at their **Thursday** morning meeting. Importantly, the pipeline refreshes the web page at intervals, so that analysis can often begin within hours of launch, with possible report refinement as final results arrive for the longer calculations that larger structures can require.

As VUStruct is not a pathogenicity predictor, but rather a tool to stimulate hypothesis generation, the computation time and cost is small in relation to the human cost of output review, structure visualization, case summary spreadsheet annotation, meeting participation, and presentation of findings in clinical context. Therefore, most of our optimization efforts are focused on the human factors. Through continuous user interface improvements to summaries and templates, in-line guidance and documentation, and pop-ups, we hope to make output increasingly valuable and easier to interpret - to an ever-broader array of scientists who could use the information – not just expert computational structural biologists and/or biochemists.

For several years, the VUStruct compute pipeline has met its weekly turnaround time without fail. On all the academic high-performance clusters on which we have worked, the massive storage resources, shared bandwidth, and competition for compute cycles can impede case runs. In contrast to an industrial setting, we do not have a Service Level Agreement (SLA) with a schedule of performance penalties for our IT team in the event of downtime. Nonetheless, the intrinsic reliability and turn-around performance of clusters is steadily improving, particularly at Vanderbilt. On the back end of the system, given the containerized structure of the pipeline, we have resorted to running on alternate hardware during cluster outages. In the web form, we strive to update the case input page with news of planned and unplanned cluster outages.

Following click of the “submit” button, for a “typical” case with 15 variants supplied in genomic coordinates, users can approximately expect:

- 5 minutes of delay for initial variant preprocessing and filtering
- 10 minutes for structure selection and job planning
- Following job launch, a preliminary analysis of results can likely take place after 6 hours or so, as many calculations will have completed.

- Following another day of calculations, the case website will be complete enough to support intensive study of results, structural visualizations, and tentative completion of the presentation worksheet. We often send the worksheet to the clinical meeting facilitator at this point, for display during our contribution.
- For the next day or so, we monitor straggler calculations for the possibility their results will further inform Friday discussions.

We believe that up to 10 such “typical” cases will soon be comfortably hosted by Vanderbilt’s recently upgraded SLURM cluster. The caveat remains that this estimate is subject to start delays, hardware outages, and other system level factors that cannot be foreseen.

We caution that the case “plan” phase (pre-launch job planning and structure selection) can take longer than 10 minutes. For variants with few covering structures, analysis can be as fast as 15 seconds per variant. When there are many large (ex. CryoEM multimer) structures for the “plan” phase to consider, we have seen worst case scenarios occur in which per-variant planning consumes 10 minutes – sometimes more. Some background for this is based in the Structure Selection algorithm (Supplemental Section). There are many opportunities for us to speed this step’s search through large structures, such as avoiding avoiding the time-consuming multi-megabyte large .cif file loads we perform to check that variants are resolved experimentally. Biopython(1) parses .cif files relatively slowly from the (often sluggish) shared filesystem by instead fast-querying pre-built databases of resolved residues for each structure that can be queried in O(constant) time for each variant.

In contrast to our ability to optimize the plan phase, the other influences on runtime are quite out of our control. When the cluster is under heavy load, we have seen calculations delayed in “pending” state for up to 24 hours before their execution begins. On occasion, they can languish longer, unfortunately.

Once jobs are launched, given the “trivial” parallelization (jobs are not interdependent), and assuming all jobs start simultaneously, all runs will be over when the slowest finishes. The slowest running jobs are typically ΔΔG runs that are requested for large structures – and we especially note slowdowns for poor quality models where there appears to be larger time requirements for relax steps which do not converge quickly. On current hardware our observation is that many ΔΔG calculations finish within the first 6 to 12 hours. Most finish within a day, and of the few stragglers, very few take longer than 2 days.

An important optimization of ΔΔG calculations is already in our code. Once a PDB chain has gone through the 20-fold “relax” process, that step is never again repeated. The final calculation fo the ΔΔG is very fast by comparison. (Aside from the VUStruct application, the

VUStruct “ΔΔG repository” architecture, where the relax step is not repeated, makes it quite reasonable to calculate and store all ΔΔGs for all variants of a 400-residue protein chain within a few days.)

The Pathprox algorithm has an internal kernel which randomly samples sets of variants. This code runtime scales at $O(N^2)$, N being the number of pathogenic and benign variants mapped onto the 3D structure by the algorithm. In practice, most Pathprox runs finish within a couple of hours. On large multi-chain complexes, 1.5 or 2.0 days is not unheard of.

The repeatedly run report generation code runtime scales linearly with the number of variants and selected structure. The user does not “feel” report generation, because it is invoked regularly on the back end. Until the “all jobs complete” message appears after the final report generation, the user should expect that the report display could lag the running processes on the back end by up to 30 minutes in worst case.

The successful deployment of VUStruct has depended heavily on the commitment of Vanderbilt to the ACCRE cluster, and to the many staff (both at ACCRE and the Vanderbilt Center for Structural Biology) who have supported our aspirations to build the pipeline into a public website. We hereby provide their requested official acknowledgement:

> This work leveraged the resources provided by the Vanderbilt Advanced Computing Center for Research and Education (ACCRE), a collaboratory operated by and for Vanderbilt faculty. ACCRE is comprised of over 3,000 researchers from more than 40 campus departments and six schools have utilized ACCRE for their research and education programs. The ACCRE computing cluster currently boasts over 16,000 processor cores and continues to expand.

References

1. Cock PJA, Antao T, Chang JT, Chapman BA, Cox CJ, Dalke A, et al. Biopython: freely available Python tools for computational molecular biology and bioinformatics. Bioinformatics. 2009 Jun 1;25(11):1422–3.
