# Supplementary material for: VUStruct: A compute pipeline for high throughput and personalized structural biology
Source: PLoS Comput Biol. 2026 May 4;22(5):e1014183. doi: 10.1371/journal.pcbi.1014183 (PMC13160433; doi:10.1371/journal.pcbi.1014183)
Supplement: S4 Text — (PDF) [file pcbi.1014183.s004.pdf]

S4 VUStruct Supplemental Information

Towards Quantitative Assessment of Clinical Utility

VUStruct presents discrete calculations, all of which have been validated in their source citations. VUStruct's results and integrated visualization point to mechanistic hypotheses about the molecular basis of disease. Thus, VUStruct adds helpful complementary information to the landscape of raw pathogenicity scores which are common in clinical genetics. Our lived experience is that while these nascent hypotheses cannot be summarized into a single ranking for each patient case, VUStruct results often inform, support, or reject the genes selected for further discussion at clinician meetings over the last 8 years

In rare genetic diseases, the rarity of the diseases itself precludes a robust "ground truth" against which to evaluate VUStruct, even when diagnoses are made. Moreover, decisions about candidate genes are made not by VUStruct alone, but by experts reflecting on VUStruct outputs in context of other inputs from the clinic and other data sources. Restated, the calculation results are evaluated in context of many additional soft inputs. Often VUSs with compelling VUStruct calculations are eliminated for lack of clinical overlap with patient phenotype. Thus, a universe of inputs against which we might quantitatively benchmark VUStruct is unclear.

Nonetheless, a more rigorous quantitative assessment of and accounting for VUStruct's clinical utility is clearly desirable, as is the development of standardized data formats, workflows, and approaches for addressing rare undiagnosed diseases' genetic etiology.

Since 2017, VUStruct pipeline calculation results have informed the structural biologists who have consulted Vanderbilt's UDN clinical team at the end of weekly case discussions. Through Sept. 2025, these consultations have taken place for ~250 UDN cases (in addition to cases local to the Vanderbilt clinic). While many of these cases are still being evaluated, Vanderbilt's UDN team has separately recorded 135 unique case IDs as "solved" at the central Harvard-hosted UDN Gateway repository. Of these 135 "solved" cases, 93 diagnoses are annotated as "Certain" and 34 as "Highly Likely"

As UDN policies protect Patient Health Information (PHI) and the case data are expressly for the internal use of UDN, we can provide only summary statistics. The "solved" UDN cases exhibit complicated etiologies. Indeed, 19 of the 135 "solved" cases fail to identify a single causal gene. Among the 116 cases with single genes implicated, VUStruct pipeline-based structural biology consultations were requested for 39 of these patient cases. (Other cases were solved through other operational pathways in the clinic.)

To retroactively illustrate VUStruct's contribution to hypothesis generation, we ran the *current* VUStruct pipeline again with the gene and variant list from the 39 "solved" cases on which we had consulted. The frequencies of calculation "hits" (see S1. Clinical Case Support and Variant Interpretation) are recorded in Table S4.1 below.

Table S4: Percentage of 39 “solved” patient cases at the Vanderbilt UDN in which VUStruct calculation “hits” were observed for the VUSs recorded as causal. All methods are cited in the main paper text.

**VUStruct-integrated calculation run on the causal VUSs**	**“Hit” definition (see S1. Clinical Case Support and Variant Interpretation)**	**Percentage of patient cases in which a VUStruct “hit” was observed on the reported VUS.**
Rosetta ΔΔGCartesian	|ΔΔG| > 2.0 REUs observed for variant with high resolution experimental structure, high confidence Alphafold, and/or high sequence identity homology models – ideally accompanied by a biophysical explanation aided by visualization (ex. Loss of hydrogen bond, steric clash, etc).	74%
PathProx (ClinVar vs gnomAD)	Positive Pathprox score with high AUC, coupled with structure visualization of pathogenic ClinVar positions vs. gnomAD.	21%
COSMIS	Z-score < -2.0	8%
ScanNet PPI predictor	Variant predicted (>50%) to participate in a protein-protein interaction	15%
DiGePred and DIEP*	Digenic Disease Predication	<5%*
Pesto PPI	Variant predicted (>50%) to be in a PPI	13%
Pesto DNA interaction	predicted (>50%) to interact with DNA	5%
Pesto ION interaction	>50% probability to interact with ion(s)	5%
Pesto ligand interaction	>50% probability to interact with ligand(s)	3%
Pesto Lipid interaction	>50% probability to interact with lipid(s)	8%
MusiteDeep	Post-translational modification (PTM) predicted within 8Å of variant, confirmed with visualization	13%
AlphaMissense	Score > 0.564	39%
Case VUSs with 2+ hits		67%
Case VUSs with 3+ hits		23%

* DiGePred and DIEP “hit rates” are not included, because this table was generated for cases where only single genes are recorded as causal. While we share great enthusiasm for the future of oligogenic disease predictors with our clinic partners, we anecdotally find them to be informative in under 5% of patient cases.

UDN candidate variants analyzed by VUStruct have high percentages of "hits" for potential disruption the various functional mechanisms tested. This is unsurprising, as the variants sent through VUStruct are pre-screened by the UDN genetics team for rarity in the general population and high traditional pathogenicity scores. Our review of our free-text case discussion notes suggests that VUStruct can add information for at least 80% of the coding VUSs that are actively discussed by the MDs.

While the table's end-to-end statistics demonstrate the relevance of VUStruct to case analysis, more specific quantification of VUStruct's impact to every week's case analysis is complicated by several factors. The anecdotal notes that document the guidance of VUStruct are variable depending on the structural biology consultant giving the report. In addition, when VUStruct supports retention of a gene for additional clinical testing, the clinic's workflow does not include a standing loopback to structural biology. Following its mention in the meeting summary, our structural biology team may not be involved again unless we are specifically asked to generate a 3D representation or contribute additional information for publication purposes.

Importantly, the integration of structural biology into clinical case discussion does not end with VUStruct. For cases involving genomic level structural changes, complex insertions, deletions, or stop gains, we give estimates of lost or impacted domains, and we offer modeling services to supplement the weekly consultations.
