# Supplementary material for: VUStruct: A compute pipeline for high throughput and personalized structural biology
Source: PLoS Comput Biol. 2026 May 4;22(5):e1014183. doi: 10.1371/journal.pcbi.1014183 (PMC13160433; doi:10.1371/journal.pcbi.1014183)
Supplement: S1 File — (PDF) [file pcbi.1014183.s005.pdf]

S5 Members of the Undiagnosed Diseases Network (Version: 10.11.2024)

Full Name	Affiliation	Email
Alyssa A. Tran	BCM Clinical	alyssat@bcm.edu
Arjun Tarakad	BCM Clinical	tarakad@bcm.edu
Ashok Balasubramanyam	BCM Clinical	ashokb@bcm.edu
Brendan H. Lee	BCM Clinical	blee@bcm.edu
Carlos A. Bacino	BCM Clinical	cbacino@bcm.edu
Daryl A. Scott	BCM Clinical	dscott@bcm.edu
Elaine Seto	BCM Clinical	esseto@bcm.edu
Gary D. Clark	BCM Clinical	gdclark@texaschildrens.org
Hongzheng Dai	BCM Clinical	Hongzheng.Dai@bcm.edu
Hsiao-Tuan Chao	BCM Clinical	hc140077@bcm.edu
Ivan Chinn	BCM Clinical	Ivan.Chinn@bcm.edu
James P. Orengo	BCM Clinical	james.orengo@bcm.edu
Jennifer E. Posey	BCM Clinical	Jennifer.Posey@bcm.edu
Jill A. Rosenfeld	BCM Clinical	mokry@bcm.edu
Kim Worley	BCM Clinical	kworley@bcm.edu
Lindsay C. Burrage	BCM Clinical	burrage@bcm.edu
Lisa T. Emrick	BCM Clinical	emrick@bcm.edu
Lorraine Potocki	BCM Clinical	lpotocki@bcm.edu
Monika Weisz Hubshman	BCM Clinical	hubshman@bcm.edu
Richard A. Lewis	BCM Clinical	rlewis@bcm.edu
Ronit Marom	BCM Clinical	ronit.marom@bcm.edu
Seema R. Lalani	BCM Clinical	seemal@bcm.edu
Shamika Ketkar	BCM Clinical	ketkar@bcm.edu
Tiphanie P. Vogel	BCM Clinical	tiphanie.vogel@bcm.edu
William J. Craigen	BCM Clinical	wcraigen@bcm.edu
Lauren Blieden	BCM Clinical	Lauren.Blieden@bcm.edu
Jared Sninsky	BCM Clinical	Jared.Sninsky@bcm.edu
Hugo J. Bellen	BCM MOSC	hbellen@bcm.edu
Michael F. Wangler	BCM MOSC	mw147467@bcm.edu
Oguz Kanca	BCM MOSC	Oguz.Kanca@bcm.edu
Shinya Yamamoto	BCM MOSC	yamamoto@bcm.edu
Christine M. Eng	BCM Sequencing	ceng@bcm.edu
Patricia A. Ward	BCM Sequencing	pward@bcm.edu
Pengfei Liu	BCM Sequencing	pliu@baylorgenetics.com
Adeline Vanderver	CHOP	vandervera@chop.edu

Cara Skraban	CHOP	skrabanc@chop.edu
Edward Behrens	CHOP	behrens@chop.edu
Gonench Kilich	CHOP	kilichg@chop.edu
Kathleen Sullivan	CHOP	sullivank@chop.edu
Kelly Hassey	CHOP	hasseyk@chop.edu
Ramakrishnan Rajagopalan	CHOP	rajagopalanr@chop.edu
Rebecca Ganetzky	CHOP	ganetzkyr@chop.edu
Vishnu Cuddapah	CHOP	cuddapahv@chop.edu
Anna Raper	CHOP/UPenn	rapera@pennmedicine.upenn.edu
Daniel J. Rader	CHOP/UPenn	rader@pennmedicine.upenn.edu
Giorgio Sirugo	CHOP/UPenn	Giorgio.Sirugo@pennmedicine.upenn.edu
Vaidehi Jobanputra	Columbia	vj2004@cumc.columbia.edu
Allyn McConkie-Rosell	Duke	allyn.mcconkie@duke.edu
Kelly Schoch	Duke	kelly.schoch@duke.edu
Mohamad Mikati	Duke	mohamad.mikati@duke.edu
Nicole M. Walley	Duke	nicole.walley@duke.edu
Rebecca C. Spillmann	Duke	rebecca.crimian@duke.edu
Vandana Shashi	Duke	vandana.shashi@duke.edu
Alan H. Beggs	Harvard	beggs@enders.tch.harvard.edu
Calum A. MacRae	Harvard	camacrae@bics.bwh.harvard.edu
David A. Sweetser	Harvard	dsweetser@partners.org
Deepak A. Rao	Harvard	darao@bwh.harvard.edu
Edwin K. Silverman	Harvard	ed.silverman@channing.harvard.edu
Elizabeth L. Fieg	Harvard	efieg@bwh.harvard.edu
Frances High	Harvard	fhigh@partners.org
Gerard T. Berry	Harvard	gerard.berry@childrens.harvard.edu
Ingrid A. Holm	Harvard	ingrid.holm@childrens.harvard.edu
J. Carl Pallais	Harvard	Juan.Pallais@mgh.harvard.edu
Joan M. Stoler	Harvard	joan.stoler@childrens.harvard.edu
Joseph Loscalzo	Harvard	jloscalzo@partners.org
Lance H. Rodan	Harvard	lance.rodan@childrens.harvard.edu
Laurel A. Cobban	Harvard	lcobban@bwh.harvard.edu
Lauren C. Briere	Harvard	lbriere@partners.org
Matthew Coggins	Harvard	mcoggins@bwh.harvard.edu
Melissa Walker	Harvard	walker.melissa@mgh.harvard.edu
Richard L. Maas	Harvard	maas@genetics.med.harvard.edu
Susan Korrick	Harvard	skorrick@bwh.harvard.edu
Jessica Douglas	Harvard	Jessica.Douglas@childrens.harvard.edu
AudreyStephannie C. Maghiro	Harvard DMCC	audreystephannie_maghiro@hms.harvard.edu
Cecilia Esteves	Harvard DMCC	cecilia_esteves@hms.harvard.edu
Emily Glanton	Harvard DMCC	Emily_Glanton@hms.harvard.edu

Isaac S. Kohane	Harvard DMCC	isaac_kohane@hms.harvard.edu
Kimberly LeBlanc	Harvard DMCC	kimberly_leblanc@hms.harvard.edu
Rachel Mahoney	Harvard DMCC	rachel_mahoney@hms.harvard.edu
Shamil R. Sunyaev	Harvard DMCC	ssunyaev@hms.harvard.edu
Shilpa N. Kobren	Harvard DMCC	Shilpa_Kobren@hms.harvard.edu
Brett H. Graham	IU	bregraha@iu.edu
Erin Conboy	IU	econboy@iu.edu
Francesco Vetrini	IU	fvetrini@iu.edu
Kayla M. Treat	IU	ktreat@iuhealth.org
Khurram Liaqat	IU	kliaqat@iu.edu
Lili Mantcheva	IU	lmantche@iu.edu
Stephanie M. Ware	IU	stware@iu.edu
Breanna Mitchell	Mayo Clinic	Mitchell.Breanna@mayo.edu
Brendan C. Lanpher	Mayo Clinic	lanpher.brendan@mayo.edu
Devin Oglesbee	Mayo Clinic	oglesbee.devin@mayo.edu
Eric Klee	Mayo Clinic	klee.eric@mayo.edu
Filippo Pinto e Vairo	Mayo Clinic	vairo.filippo@mayo.edu
Ian R. Lanza	Mayo Clinic	lanza.ian@mayo.edu
Kahlen Darr	Mayo Clinic	Darr.Kahlen@mayo.edu
Lindsay Mulvihill	Mayo Clinic	mulvihill.lindsay@mayo.edu
Lisa Schimmenti	Mayo Clinic	Schimmenti.Lisa@mayo.edu
Queenie Tan	Mayo Clinic	Tan.KhoonGheeQueenie@mayo.edu
Surendra Dasari	Mayo Clinic	dasari.surendra@mayo.edu
Adriana Rebelo	Miami	arebelo@med.miami.edu
Carson A. Smith	Miami	carsonsmith@med.miami.edu
Deborah Barbouth	Miami	dbarbouth@miami.edu
Guney Bademci	Miami	g.bademci@miami.edu
Joanna M. Gonzalez	Miami	jmg442@miami.edu
Kumarie Latchman	Miami	kxl604@med.miami.edu
LéShon Peart	Miami	L.peart@med.miami.edu
Mustafa Tekin	Miami	mtekin@miami.edu
Nicholas Borja	Miami	nborja@med.miami.edu
Stephan Zuchner	Miami	szuchner@miami.edu
Stephanie Bivona	Miami	sab355@miami.edu
Willa Thorson	Miami	wthorson@miami.edu
Herman Taylor	Morehouse DMCC	htaylor@msm.edu
Andrea Gropman	NIH UDP	agropman@childrensnational.org
Barbara N. Pusey Swerdzewski	NIH UDP	barbara.pusey@nih.gov
Camilo Toro	NIH UDP	toroc@mail.nih.gov
Colleen E. Wahl	NIH UDP	colleen.wahl@nih.gov
Donna Novacic	NIH UDP	donna.novacic@nih.gov

Ellen F. Macnamara	NIH UDP	ellen.macnamara@nih.gov
John J. Mulvihill	NIH UDP	johmulvihill@gmail.com
Maria T. Acosta	NIH UDP	acostam@nhgri.nih.gov
Precilla D'Souza	NIH UDP	precilla.d'souza@nih.gov
Valerie V. Maduro	NIH UDP	vbraden@mail.nih.gov
Ben Afzali	NIH UDP, NHGRI	ben.afzali@nih.gov
Ben Solomon	NIH UDP, NHGRI	solomonb@mail.nih.gov
Cynthia J. Tifft	NIH UDP, NHGRI	ctifft@nih.gov
David R. Adams	NIH UDP, NHGRI	david.adams@nih.gov
Elizabeth A. Burke	NIH UDP, NHGRI	elizabeth.burke2@nih.gov
Francis Rossignol	NIH UDP, NHGRI	francis.rossignol@nih.gov
Heidi Wood	NIH UDP, NHGRI	heidi.wood@nih.gov
Jiayu Fu	NIH UDP, NHGRI	fuj6@mail.nih.gov
Joie Davis	NIH UDP, NHGRI	jdavis@niaid.nih.gov
Leoyklang Petcharet	NIH UDP, NHGRI	petcharat.leoyklang@nih.gov
Lynne A. Wolfe	NIH UDP, NHGRI	lynne.wolfe@nih.gov
Margaret Delgado	NIH UDP, NHGRI	margaret.delgado@nih.gov
Marie Morimoto	NIH UDP, NHGRI	marie.morimoto@nih.gov
Marla Sabaii	NIH UDP, NHGRI	marla.sabaii@nih.gov
MayChristine V. Malicdan	NIH UDP, NHGRI	maychristine.malicdan@nih.gov
Neil Hanchard	NIH UDP, NHGRI	neil.hanchard@nih.gov
Orpa Jean-Marie	NIH UDP, NHGRI	orpa.jean-marie@nih.gov
Wendy Introne	NIH UDP, NHGRI	wintrone@nhgri.nih.gov
William A. Gahl	NIH UDP, NHGRI	gahlw@mail.nih.gov
Yan Huang	NIH UDP, NHGRI	yan.huang@nih.gov
Aimee Allworth	PNW	allwoa@uw.edu
Andrew Stergachis	PNW	absterga@uw.edu
Danny Miller	PNW	Danny.Miller@seattlechildrens.org
Elizabeth Blue	PNW	em27@uw.edu
Elizabeth Rosenthal	PNW	erosen@uw.edu
Elsa Balton	PNW	ebalton@medicine.washington.edu
Emily Shelkowitz	PNW	
Eric Allenspach	PNW	eric.allenspach@seattlechildrens.org
Fuki M. Hisama	PNW	fmh2@uw.edu
Gail P. Jarvik	PNW	pair@uw.edu
Ghayda Mirzaa	PNW	gmirzaa@uw.edu
Ian Glass	PNW	ianglass@uw.edu
Kathleen A. Leppig	PNW	leppig@uw.edu
Katrina Dipple	PNW	katrina.dipple@seattlechildrens.org
Mark Wener	PNW	wener@uw.edu
Martha Horike-Pyne	PNW	mpyne@medicine.washington.edu

Michael Bamshad	PNW	mbamshad@uw.edu
Peter Byers	PNW	pbyers@uw.edu
Sam Sheppeard	PNW	samshep@uw.edu
Sirisak Chanprasert	PNW	sirisc@uw.edu
Virginia Sybert	PNW	flk01@uw.edu
Wendy Raskind	PNW	wendyrun@uw.edu
Nitsuh K. Dargie	PNW	nitsuhk@medicine.washington.edu
Beth A. Martin	Stanford	martinb@stanford.edu
Chloe M. Reuter	Stanford	creuter@stanfordhealthcare.org
Devon Bonner	Stanford	devonbonner@stanfordhealthcare.org
Elijah Kravets	Stanford	ekravets@stanford.edu
Holly K. Tabor	Stanford	hktabor@stanford.edu
Jacinda B. Sampson	Stanford	jacindas@stanford.edu
Jason Hom	Stanford	jasonhom@stanford.edu
Jennefer N. Kohler	Stanford	jkohler@stanfordhealthcare.org
Jonathan A. Bernstein	Stanford	Jon.Bernstein@stanford.edu
Kevin S. Smith	Stanford	kssmith@stanford.edu
Matthew T. Wheeler	Stanford	wheelerm@stanford.edu
Meghan C. Halley	Stanford	mhalley@stanford.edu
Page C. Goddard	Stanford	pgoddard@stanford.edu
Paul G. Fisher	Stanford	pfisher@stanford.edu
Rachel A. Ungar	Stanford	raungar@stanford.edu
Raquel L. Alvarez	Stanford	raquela1@stanford.edu
Shruti Marwaha	Stanford	mshruti@stanford.edu
Terra R. Coakley	Stanford	tcoakley@stanford.edu
Euan A. Ashley	Stanford DMCC	Euan@stanford.edu
Ali Al-Beshri	UAB	asabeshri@uabmc.edu
Anna Hurst	UAB	acehurst@uab.edu
Bruce Korf	UAB	bkorf@uab.uabmc.edu
Kaitlin Callaway	UAB	kcallaway@uabmc.edu
Martin Rodriguez	UAB	rodriguez@uabmc.edu
Tammi Skelton	UAB	tlskelton@uabmc.edu
Andrew B. Crouse	UAB DMCC	acrouse@uab.edu
Jordan Whitlock	UAB DMCC	jbarham3@uab.edu
Mariko Nakano-Okuno	UAB DMCC	marikonk@uab.edu
Matthew Might	UAB DMCC	might@uab.edu
William E. Byrd	UAB DMCC	webyrd@gmail.com
Changrui Xiao	UCI/CHOC	changrx@hs.uci.edu
Eric Vilain	UCI/CHOC	evilain@hs.uci.edu
Jose Abdenur	UCI/CHOC	JAbdenur@choc.org
Kathyrn Singh	UCI/CHOC	kesingh@hs.uci.edu

Rebekah Barrick	UCI/CHOC	rebekah.barrick@choc.org
Sanaz Attaripour	UCI/CHOC	sattarip@hs.uci.edu
Suzanne Sandmeyer	UCI/CHOC	sbsandme@hs.uci.edu
Tahseen Mozaffar	UCI/CHOC	mozaffar@hs.uci.edu
Albert R. La Spada	UCI/CHOC	alaspada@uci.edu
Elizabeth C. Chao	UCI/CHOC	ecchao@uci.edu
Maija-Rikka Steenari	UCI/CHOC	msteenari@choc.org
Alden Huang	UCLA	AYHuang@mednet.ucla.edu
Brent L. Fogel	UCLA	bfogel@ucla.edu
Esteban C. Dell'Angelica	UCLA	edellangelica@mednet.ucla.edu
George Carvalho	UCLA	GCarvalhoNeto@mednet.ucla.edu
Julian A. Martínez-Agosto	UCLA	julianmartinez@mednet.ucla.edu
Manish J. Butte	UCLA	mbutte@mednet.ucla.edu
Martin G. Martin	UCLA	mmartin@mednet.ucla.edu
Naghmeh Dorrani	UCLA	ndorrani@mednet.ucla.edu
Neil H. Parker	UCLA	nhparker@mednet.ucla.edu
Rosario I. Corona	UCLA	rcoronadelafuente@mednet.ucla.edu
Stanley F. Nelson	UCLA	snelson@ucla.edu
Yigit Karasozen	UCLA	Ykarasozen@mednet.ucla.edu
Aaron Quinlan	University of Utah	aquinlan@genetics.utah.edu
Alistair Ward	University of Utah	alistairnward@gmail.com
Ashley Andrews	University of Utah	ashley.andrews@hsc.utah.edu
Corrine K. Welt	University of Utah	cwelt@u2m2.utah.edu
Dave Viskochil	University of Utah	dave.viskochil@hsc.utah.edu
Erin E. Baldwin	University of Utah	erin.baldwin@hsc.utah.edu
John Carey	University of Utah	john.carey@hsc.utah.edu
Justin Alvey	University of Utah	justin.alvey@hsc.utah.edu
Laura Pace	University of Utah	laura.pace@hsc.utah.edu
Lorenzo Botto	University of Utah	lorenzo.botto@hsc.utah.edu
Nicola Longo	University of Utah	nicola.longo@hsc.utah.edu
Paolo Moretti	University of Utah	paolo.moretti@hsc.utah.edu
Rebecca Overbury	University of Utah	rebecca.overbury@hsc.utah.edu
Russell Butterfield	University of Utah	russell.butterfield@hsc.utah.edu
Steven Boyden	University of Utah	steven.boyden@genetics.utah.edu
Thomas J. Nicholas	University of Utah	thomas.nicholas@utah.edu
Matt Velinder	University of Utah	mvelinder@frameshift.io
Gabor Marth	University of Utah DMCC	gmarth@genetics.utah.edu
Pinar Bayrak-Toydemir	University of Utah/ARUP	pinar.bayrak-toydemir@arup.com
Rong Mao	University of Utah/ARUP	rong.mao@aruplab.com

Monte Westerfield	UO MOSC	monte@uoneuro.uoregon.edu
Brian Corner	Vanderbilt	brian.corner@vumc.org
John A. Phillips III	Vanderbilt	John.a.phillips@vumc.org
Kimberly Ezell	Vanderbilt	kimberly.ezell@vumc.org
Lynette Rives	Vanderbilt	lynette.c.rives@vumc.org
Rizwan Hamid	Vanderbilt	rizwan.hamid@vumc.org
Serena Neumann	Vanderbilt	serena.neumann@vumc.org
Ashley McMinn	Vanderbilt	ashley.mcminn@vumc.org
Joy D. Cogan	Vanderbilt	joy.cogan@vumc.org
Thomas Cassini	Vanderbilt	thomas.a.cassini@vumc.org
Alex Paul	WUSTL Clinical	alex.paul@wustl.edu
Dana Kiley	WUSTL Clinical	dana.kiley@wustl.edu
Daniel Wegner	WUSTL Clinical	danieljwegner@wustl.edu
Erin McRoy	WUSTL Clinical	e.hediger@wustl.edu
Jennifer Wambach	WUSTL Clinical	wambachj@wustl.edu
Kathy Sisco	WUSTL Clinical	siscok@wustl.edu
Patricia Dickson	WUSTL Clinical	pdickson@wustl.edu
F. Sessions Cole	WUSTL DMCC	fcole@wustl.edu
Dustin Baldridge	WUSTL MOSC	dbaldri@wustl.edu
Jimann Shin	WUSTL MOSC	shinji@wustl.edu
Lilianna Solnica-Krezel	WUSTL MOSC	solnical@wustl.edu
Stephen Pak	WUSTL MOSC	stephen.pak@email.wustl.edu
Timothy Schedl	WUSTL MOSC	ts@wustl.edu
Hector Rodrigo Mendez	Stanford	mendezh@stanford.edu
Brianna Tucker	Stanford	bmtucker@stanford.edu
Beatriz Anguiano	Stanford	banguian@stanford.edu
Mia Levanto	Stanford	mlevanto@stanford.edu
Suha Bachir	Stanford	sbachir@stanford.edu
Laurens Wiel	Stanford	lvdwiel@stanford.edu
Stephen B Montgomery	Stanford	smontgom@stanford.edu
Tanner D Jensen	Stanford	tannerj@stanford.edu
John E. Gorzynski	Stanford	jgorz@stanford.edu
Sara Emami	Stanford	slemami@stanford.edu
Laura Keehan	Stanford	keehan@stanford.edu
Jennifer Schymick	Stanford	jennifer.schymick@hhs.sccgov.org
Taylor Maurer	Stanford	maurertm@stanford.edu
Alexander Miller	Stanford	atex91@stanford.edu
Andres Vargas	UCLA	AndresVargas@mednet.ucla.edu
Amanda M. Shrewsbury	UCLA	ashrewsbury@mednet.ucla.edu
Bianca E. Russell	UCLA	berussell@mednet.ucla.edu
Layal F. Abi Farraj	UCLA	LAbiFarraj@mednet.ucla.edu

Elizabeth A Worthey	UAB	eaworthey@uabmc.edu
Tarun KK Mamidi	UAB	tmamidi@uab.edu
Brandon M Wilk	UAB	brandonwilk@uabmc.edu
Rachel Li	Sanford	Rachel.Li@SanfordHealth.org
Jennifer Morgan	Sanford	Jennifer.Morgan@SanfordHealth.org
Chun-Hung Chan	Sanford	Chun-Hung.Chan@SanfordHealth.org
Paul Berger	Sanford	Paul.berger@sanfordhealth.org
Mohamad Saifeddine	Sanford	Mohamad.Saifeddine@SanfordHealth.org
Isum Ward	Sanford	Isum.Ward@SanfordHealth.org
Jason Schend	Sanford	Jason.Schend@SanfordHealth.org
Megan Bell	Sanford	Megan.bell@sanfordhealth.org
Dr. Francisco Bustos velasq	Sanford	Francisco.Bustos@sanfordhealth.org
Taylor Beagle	Sanford	Taylor.Beagle@SanfordHealth.org
Miranda Leitheiser	Sanford	Miranda.Leitheiser@SanfordHealth.org
Runjun Kumar	WUSTL Clinical	rdkumar@uw.edu
Donald Basel	MCW-CW	dbasel@mcw.edu
Michael Muriello	MCW-CW	mmuriello@mcw.edu
Brett Bordini	MCW-CW	bbordini@mcw.edu
Michael Zimmermann	MCW-CW	mtzimmermann@mcw.edu
Abdul Elkadri	MCW-CW	AElKadri@mcw.edu
James Verbsky	MCW-CW	jverbsky@mcw.edu
Julie McCarrier	MCW-CW	jmccarrier@mcw.edu
